# Quality standards in proteomics research facilities: Common standards and quality procedures are essential for proteomics facilities and their users

**DOI:** 10.15252/embr.202152626

**Published:** 2021-05-19

**Authors:** Cristina Chiva, Teresa Mendes Maia, Christian Panse, Karel Stejskal, Thibaut Douché, Mariette Matondo, Damarys Loew, Dominic Helm, Mandy Rettel, Karl Mechtler, Francis Impens, Paolo Nanni, Anna Shevchenko, Eduard Sabidó

**Affiliations:** ^1^ Centre de Regulació Genòmica Barcelona Institute of Science and Technology (BIST) Barcelona Spain; ^2^ Univeristat Pompeu Fabra Barcelona Spain; ^3^ VIB Proteomics Core VIB Ghent Belgium; ^4^ VIB‐UGent Center for Medical Biotechnology VIB Ghent Belgium; ^5^ Department of Biomolecular Medicine Ghent University Ghent Belgium; ^6^ Functional Genomics Center Zurich University/ETH Zurich Zurich Switzerland; ^7^ Swiss Institute of Bioinformatics Lausanne Switzerland; ^8^ Research Institute of Molecular Pathology (IMP) Vienna Biocenter (VBC) Vienna Austria; ^9^ IMBA Institute of Molecular Biotechnology of the Austrian Academy of Sciences Vienna Biocenter (VBC) Vienna Austria; ^10^ Institut Pasteur, Proteomics Platform, Mass Spectrometry for Biology Unit, USR CNRS 2000 MSBio Unit CNRS Paris France; ^11^ Institut Curie, Centre de Recherche, Laboratoire de Spectrométrie de Masse Protéomique PSL Research University Paris cedex 05 France; ^12^ Proteomics Core Facility European Molecular Biology Laboratory Heidelberg Germany; ^13^ MS based Protein analysis Unit Genomics Proteomics Core Facilities DKFZ Heidelberg Germany; ^14^ Gregor Mendel Institute (GMI) Austrian Academy of Sciences, Vienna BioCenter 7 (VBC) Vienna Austria; ^15^ Max Planck Institute of Molecular Cell Biology and Genetics Dresden Germany

## Abstract

Proteomics research infrastructures and core facilities within the Core for Life alliance advocate for community policies for quality control to ensure high standards in proteomics services.

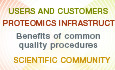

Core facilities and research infrastructures have become an essential part of the scientific ecosystem. In the field of proteomics, national and international networks and research platforms have been established during the past decade that are supposed to set standards for high‐quality services, promote an exchange of professional information, and enable access to cutting‐edge, specialized proteomics technologies. Either centralized or distributed, these national and international proteomics infrastructures and technology platforms are generating massive amounts of data for the research community, and support a broad range of translational, computational and multi‐omics initiatives and basic research projects.

By delegating part of their work to these services, researchers expect that the core facility adjusts their analytical protocols appropriately for their project to acquire data conforming best research practice of the scientific community. The implementation of quality assessment measures and commonly accepted quality controls in data generation is therefore crucially important for proteomics research infrastructures and the scientists who rely on them.

However, current quality control and quality assessment procedures in proteomics core facilities and research infrastructures are a motley collection of protocols, standards, reference compounds and software tools. Proteomics relies on a customized multi‐step workflow typically consisting of sample preparation, data acquisition and data processing, and the implementation of each step differs among facilities. For example, sample preparation involves enzymatic digestion of the proteins, which can be performed in‐solution, in‐gel, or on‐beads, with often different proteolytic enzymes, chemicals, and conditions among laboratories. Data acquisition protocols are often customized to the particular instrument set up, and the acquired spectra and chromatograms are processed by different software tools provided by equipment vendors, third parties or developed in‐house.

…current quality control and quality assessment procedures in proteomics core facilities and research infrastructures are a motley collection of protocols, standards, reference compounds and software tools.

Moreover, core facilities implement their own guidelines to monitor the performance and quality of the entire workflow, typically utilizing different commercially available standards such as pre‐digested cell lysates, recombinant proteins, protein mixtures, or isotopically labeled peptides. Currently, there is no clear consensus on if, when and how to perform quality control checks. There is even less quality control in walk‐in facilities, where the staff is only responsible for correct usage of the instruments and users select and execute the analytical workflow themselves. It is not surprising therefore that instrument stability and robustness of the applied analytical approach are often unclear, which compromises analytical rigor.

## Establishing standardized practices

Initiated by the HUPO Proteomics Standard Initiative (PSI) more than a decade ago, MIAPE guidelines (Minimal Information about Proteomics Experiment; Taylor *et al,* 2007) introduced common formats for sharing and reporting proteomics data, including unrestricted access to raw data at public repositories (Vizcaíno *et al,* 2014). Supported by journals’ guidelines that request the deposition of raw data into such repositories as a condition for publication, these repositories have grown into a rich resource for data mining and multi‐omics integration. However, MIAPE guidelines did not imply quality metrics and there is still no generic tool capable of independently ascertaining the technical quality of the deposited data. The importance of quality assessments for open‐access proteomics was highlighted in the Amsterdam Principles more than 10 years ago (Rodriguez *et al,* 2009), but the development of quality threshold metrics was delegated to central repositories.

A few years later, the Sydney workshop convened by the US National Cancer Institute made recommendations and formulated key principles for data quality metrics, and journal editors and reviewers were supposed to encourage or enforce their implementation in practice (Kinsinger *et al,* 2012). The corollary recognized “the need for formal comparison of methods on equal footing” thus alluding for the first time to a common quality control. More recently, recommendations for quality control metrics have indeed been included in publishing guidelines (Abbatiello *et al,* 2017).

The need for common quality assessment protocols in scientific infrastructures has also been emphasized by international research associations. The Association of Biomolecular Research Facilities (ABRF), Core Technologies for Life Sciences (CTLS), Core for Life (C4L), and the Clinical Proteomic Tumor Analysis Consortium (CPTAC), initiated discussions and development of common quality procedures, and continuously promote sharing of best practices. A recent comprehensive survey among research facilities across Europe showed that the majority of core facilities do recognize the need and importance of quality controls (Kos‐Braun *et al,* 2020). However, we believe that the issue of systematic quality procedures in proteomics infrastructures still has not received the public attention it deserves. Moreover, we maintain that community efforts toward quality control and quality assessment are not sufficiently organized to achieve systematic agreement, despite the availability of methods for the evaluation of analytical protocols, intra‐ and inter‐laboratory comparison of reproducibility, and software tools for automated monitoring of instrument performance.

A recent comprehensive survey among research facilities across Europe showed that the majority of core facilities do recognize the need and importance of quality controls.

## Common quality control procedures

**Figure 1:**
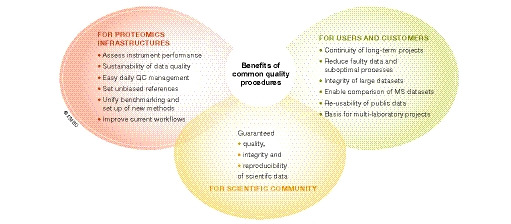
Benefits of common quality procedures Main benefits of the implementation of common quality procedures in proteomics research infrastructures and core facilities for the users and customers, the scientific community, and the infrastructures themselves.

Common quality control procedures in proteomics core facilities ensure technical quality, reproducibility, comparability, and data integrity. A representative example of how these benefit the coordinated work of several proteomics units is the dissection and validation of a SARS protein interaction map (Gordon *et al,* 2020). Common quality controls foster reuse of resources, protect against bias in experimental design and improve daily routines (Fig 1). Systematic assessment of instrument performance, early recognition of poor‐quality data, and monitoring carry‐over and background signals enable long‐term robustness and reproducibility of the proteomics workflow and leverage the impact of aging instruments or turnaround of the laboratory staff.

Quality control procedures should be generic and flexible and support diverse workflows and instrumental platforms. Core facilities and research infrastructures are technology hubs and their operations are not, and should not, be limited to routine analytical measurements. Diversity of model organisms, scales, and research goals of the scientific community generate numerous project‐specific protocols and great variability of workflows. Instrumentation platforms and analytical software will also remain diverse and heterogeneous in the foreseeable future. The choice of mass spectrometry equipment is often not only defined by scientific requirements but also influenced by the availability of funding and results in a collection of instruments of different generations, types, and vendors within the same facility.

Core facilities and research infrastructures are technology hubs and their operations are not, and should not, be limited to routine analytical measurements.

Quality procedures should be therefore organized into a framework that accommodates these diverse workflows and instrumental platforms. Such a framework should rely on common commercially available protein and peptide standards that—alone or spiked into the samples—are systematically analyzed for values relevant for quality control, such as the number identified of proteins, retention time and intensity of the peptide chromatographic peaks, ratio, or fold change of endogenous and isotopically labeled reference peptides. These repeated test runs would document the analytical performance of the entire workflow applied to an individual sample or sample batch. The information would be highly valuable for detecting random failures, monitoring instrument stability, and ensuring the reproducibility of repeated analyses, but also for continuous methods optimization and developing new methods. Moreover, common quality control parameters should be submitted to repositories along with the raw result data as required by MIAPE guidelines.

… common quality control parameters should be submitted to repositories along with the raw result data as required by MIAPE guidelines.

The frequent analysis of standard samples would also help to generate laboratory‐based average references that monitor the performance drift of instruments and indicate whether the settings applied to analytical and computational workflow are optimal or would need readjustment. Such records could help to diagnose instrument malfunction and to test the performance of new instruments. In the future, software tools could be integrated with both instruments and reference data repositories to streamline the collection and management of quality control values. The aforementioned procedures could be a step towards ISO (International Organization for Standardization) or other quality certifications for core facilities and research infrastructures that require it.

## The role of funders and technology providers

The development and implementation of common quality management schemes pose a challenge for the entire proteomics community and require support from technology providers and funding agencies. The proteomics community must therefore define a common set of quality parameters, standards, controlled vocabulary, and generic file formats to support collective testing and anonymized evaluation of the results. They should also work with technology providers to implement quality checks in vendors’ software. Last but not least, the community should approach national and international funding bodies to raise awareness of the importance of common quality control procedure in order to secure their financial support.

Research infrastructures and core facilities are in the position to drive initiatives that require extensive collaboration and concerted efforts. Within the Core for Life alliance (Meder *et al,* 2016; Lippens *et al,* 2019) (https://coreforlife.eu), our proteomics laboratories advocate for community policies for quality control procedures to ensure the high standards in proteomics services. Among other initiatives, we have developed and endorsed the QCloud tool as a cross‐platform open‐source quality control software for systematic monitoring of instrument performance (Chiva *et al,* 2018; Olivella *et al,* 2021). However, there is a further need in developing automated, user‐friendly, and flexible routines suitable for inter‐laboratory collection of quality data that satisfy data protection requirements and remain affordable for the broader research community. These and other practical measures require understanding and support of the entire proteomics community, funding, and publishing bodies.

## References

[embr202152626-bib-0001] Abbatiello S , Ackermann BL , Borchers C , Bradshaw RA , Carr SA , Chalkley R , Choi M , Deutsch E , Domon B , Hoofnagle AN *et al* (2017) New guidelines for publication of manuscripts describing development and application of targeted mass spectrometry measurements of peptides and proteins. Mol Cell Proteomics 16: 327–328 28183812 10.1074/mcp.E117.067801 PMC5340997

[embr202152626-bib-0002] Chiva C , Olivella R , Borràs E , Espadas G , Pastor O , Solé A , Sabidó E (2018) QCloud: A cloud‐based quality control system for mass spectrometry‐based proteomics laboratories. PLoS One 13: e0189209 29324744 10.1371/journal.pone.0189209 PMC5764250

[embr202152626-bib-0003] Gordon DE , Jang GM , Bouhaddou M , Xu J , Obernier K , White KM , O’Meara MJ , Rezelj VV , Guo JZ , Swaney DL *et al* (2020) A SARS‐CoV‐2 protein interaction map reveals targets for drug repurposing. Nature 583: 459–468 32353859 10.1038/s41586-020-2286-9 PMC7431030

[embr202152626-bib-0004] Kinsinger CR , Apffel J , Baker M , Bian X , Borchers CH , Bradshaw R , Brusniak M‐Y , Chan DW , Deutsch EW , Domon B *et al* (2012) Recommendations for mass spectrometry data quality metrics for open access data (corollary to the Amsterdam Principles). J Proteome Res 11: 1412–1419 22053864 10.1021/pr201071t PMC3272102

[embr202152626-bib-0005] Kos‐Braun IC , Gerlach B , Pitzer C (2020) A survey of research quality in core facilities. Elife 9: e62212 33241998 10.7554/eLife.62212 PMC7714392

[embr202152626-bib-0006] Lippens S , D'Enfert C , Farkas L , Kehres A , Korn B , Morales M , Pepperkok R , Premvardhan L , Schlapbach R , Tiran A *et al* (2019) One step ahead: Innovation in core facilities. EMBO Rep 20: e48017 30872318 10.15252/embr.201948017 PMC6446189

[embr202152626-bib-0007] Meder D , Morales M , Pepperkok R , Schlapbach R , Tiran A , Van Minnebruggen G (2016) Institutional core facilities: prerequisite for breakthroughs in the life sciences: core facilities play an increasingly important role in biomedical research by providing scientists access to sophisticated technology and expertise. EMBO Rep 17: 1088–1093 27412771 10.15252/embr.201642857 PMC4967956

[embr202152626-bib-0008] Olivella R , Chiva C , Serret M , Mancera D , Cozzuto L , Hermoso A , Borràs E , Espadas G , Morales J , Pastor O *et al* (2021) QCloud2: an improved cloud‐based quality‐control system for mass‐spectrometry‐based proteomics laboratories. J Proteome Res 20: 2010–2013 33724836 10.1021/acs.jproteome.0c00853

[embr202152626-bib-0009] Rodriguez H , Snyder M , Uhlén M , Andrews P , Beavis R , Borchers C , Chalkley RJ , Cho SY , Cottingham K , Dunn M *et al* (2009) Recommendations from the 2008 international summit on proteomics data release and sharing policy: the Amsterdam principles. J Proteome Res 8: 3689–3692 19344107 10.1021/pr900023z PMC2742685

[embr202152626-bib-0010] Taylor CF , Paton NW , Lilley KS , Binz P‐A , Julian RK , Jones AR , Zhu W , Apweiler R , Aebersold R , Deutsch EW *et al* (2007) The minimum information about a proteomics experiment (MIAPE). Nat Biotechnol 25: 887–893 17687369 10.1038/nbt1329

[embr202152626-bib-0011] Vizcaíno JA , Deutsch EW , Wang R , Csordas A , Reisinger F , Ríos D , Dianes JA , Sun Z , Farrah T , Bandeira N *et al* (2014) ProteomeXchange provides globally coordinated proteomics data submission and dissemination. Nat Biotechnol 32: 223–226 24727771 10.1038/nbt.2839 PMC3986813

